# Measuring informal workplace learning outcomes in residency training: a validation study

**DOI:** 10.1186/s12909-023-04529-1

**Published:** 2023-08-03

**Authors:** Markus Heim, Christian M. Schulz, Frederick Schneider, Pascal O. Berberat, Martin Gartmeier, Kristina Schick

**Affiliations:** 1 Technical University of Munich, TUM School of Medicine, Department of Anesthesiology and Intensive Care Medicine, Ismaninger Straße 22, Munich, 81675 Germany; 2 Technical University Munich, TUM School of Medicine, TUM Medical Education Center, Ismaninger Straße 22, München, 81675 Germany

**Keywords:** Informal workplace learning outcomes, Residency training, Validation, Self-report measure

## Abstract

**Background:**

Informal workplace learning (WPL) has no concrete learning objective and takes place without a responsible supervisor, which makes it difficult to assess its learning outcomes. Formal learning situations, as they are known from universities or schools, do not exist in this context and make a conventional assessment of learning goals and achievements impossible. Informal learning in the workplace is of central importance, and the assessment of informal learning outcomes in medical education is an under-researched area. The aim of our study was to adapt and validate an informal WPL questionnaire (originally developed for social workers) to assess learning outcomes due to informal WPL in residency training.

**Methods:**

A total of 528 residents (*n* = 339 female; age: *M* = 29.79; *SD* = 3.37 years) completed an adapted questionnaire on informal WPL outcomes and the Freiburg Questionnaire to Assess Competencies in Medicine (i.e. medical knowledge, communication, and scholarship). Exploratory factor analysis was used to determine the underlying factor structure. The reliability of the factors was tested using McDonald’s omega, and the correlation between the factors and the three subscales of the Freiburg questionnaire was tested using Spearman’s rho correlation coefficient. To investigate construct validity, a structural equation model was calculated to examine the relationships between medical competencies and informal learning outcomes.

**Results:**

The exploratory factor analysis yielded a four-factor solution that best fit the data. The scores of all four factors (GLO-CD: generic learning outcomes—competence development, GLO-R: generic learning outcomes—reflection, JSLO: job-specific learning outcomes, and OLLO: organisational learning outcomes) showed good internal consistency (Ω ≥ .69). The structural equation model showed that "medical expertise" had an impact on all four factors of informal learning at work. “Scholarship” seemed to predict GLO-CD and GLO-R.

**Conclusions:**

Our four-factor model reveals meaningful determinants of informal WPL in relation to residency training. The instrument is therefore the first promising attempt to assess informal WPL in the broader context of medical education during residency, thus supporting its construct validity.

## Background

After graduation from medical school, physicians begin their residency training. Residency training takes place almost entirely in the workplace, where, in contrast to medical school, formal learning is largely replaced by informal workplace learning (WPL; [1, 2]). WPL occurs when physicians apply skills in the workplace they have learned in medical school to clinical practice, or when they acquire new skills they were not previously familiar with [1, 3]. The outcomes of informal WPL seem to be different from formal learning outcomes, such as academic knowledge or practical skills. In one approach to defining informal WPL outcomes, *generic learning outcomes*, *organisational learning outcomes,* and *job-specific learning outcomes* are distinguished [1]. Specifically, Kyndt et al. [1] developed and validated a questionnaire to determine informal WPL outcomes for social care workers and capture its different aspects. However, little is known about whether these informal WPL outcomes also apply to residency training. To address this research gap, we have adapted the measure proposed by Kyndt et al. [1] for implementation with young physicians in their residencies and investigated how physicians’ perceived competencies, which include “communication”, “scholarship”, and “medical expertise”, as they have learned in their undergraduate medical education, predict informal WPL outcomes in their residencies. In the following, we provide a short overview of the theoretical concept of WPL, the Questionnaire of Informal WPL Outcomes used to measure these outcomes, and the influence of prior knowledge on informal WPL outcomes.

Learning in the workplace can be described by the social constructive learning theory. Social constructivism assumes that learners learn due to experiences and apply their prior knowledge to particular (working) tasks [4]. Workplace activities foster learning through different learning opportunities, such as allowing individuals to do the work themselves, obtain feedback and collaborate with colleagues and supervisors, deal with problems and challenges, and reflect on their own behaviour at work [5]. Previous findings show that the interplay of these learning conditions in the workplace (also provided by supervisors and the organisation) can facilitate knowledge and competence acquisition among employees [5–7].

Knowledge and competence acquistion can be facilitated through diffferent WPL approaches. These approaches are differentiated into formal, informal, and non-formal WPL. Formal WPL refers to clearly defined learning conditions, including learning goals, learning “off the job” in classrooms or workshops, and having a supervisor who teaches a particular skill or topic to the resident in a planned and deliberate way [5, 8]. On the contrary, informal WPL is embedded in everyday work as it takes place, without any structure, particular learning goal, or responsible supervisor [3, 5, 7]. Nonetheless, informal WPL is crucial for becoming an expert in one’s respective medical field, since it emerges from the process of participation in activities that characterise a specific community of practice and a professional domain [9]. Previous research has assumed that almost 80% of WPL opportunities are informal [1, 2]. Finally, the concept of non-formal learning merges aspects of formal and informal WPL. Non-formal learning also occurs in the workplace during daily practice. However, even though it involves a supervisor or mentor and explicit learning goals, it is still not as structured and planned as formal learning [3]. A typical non-formal learning setting would be workplace training organised by the company (e.g. training on a new resuscitation device conducted by other colleagues or supervisors who have already been trained).

Approaches to defining informal WPL outcomes focus either on competence-related differentiation by defining knowledge, skills, and attitudes or on a holistic approach offering learning principles for different occupational levels (for a detailed summary, see [1]. Kyndt et al. [1] have defined informal WPL outcomes as “[…] sustainable changes in knowledge, skills or attitudes that result from engagement in informal and formal learning processes and that influence individuals’ present and future professional achievement and/or organizational performance” [1]. By combining the competence-related and the holistic approach, the questionnaire of informal workplace learning developed by Kyndt et al. [1] aims to investigate informal WPL outcomes of employees and to identify potential for improvement in the working environment. The generic learning outcomes (GLO) refer to outcomes for all employees of a company (i.e. the product of reflection on the daily work routine, independent of one’s own position in the team or organisation and particular profession). The organisation-level learning outcomes (OLLO) are related to subgroups or teams within an enterprise in learning how to facilitate a teamworking environment. Finally, the job-specific learning outcomes (JSLO) describes the learning process required of a particular position (i.e. to develop the competencies needed to be successful in a given profession, like surgery or radiology). This classification, as described by Kyndt et al. [1], seems to be applicable to residency training as well. In each clinical area during residency, residents are supervised by a variety of consultants with differing clinical, scientific, and didactic expertise and interests, these characteristics of supervisors properly refers to GLO and OLLO. Care has to be provided in individual medical contexts and for conditions that require residents’ medical action, which might account for JSLO. With time during residency, along with the increasing competence of the resident, the complexity of treated cases increases, as does independence from supervisors. In this process, residents become an essential part of the medical team and health care system. Additionally, desired learning outcomes are necessarily specified as learning objectives a priori. Furthermore, while employers define the conditions under which WPL occurs [5], the employees’ individual biographies, previous knowledge, and/or personal engagement in other social scopes foster their individual WPL [10]. The outcomes of informal WPL could be influenced by the prior knowledge and competencies of residents [6, 10]. Learning in general refers not only to the emergence of new knowledge, but also to the combination of new knowledge with prior knowledge [9]. Self-awareness of one’s own prior knowledge or competencies might play a crucial role in (furthering) the development of competencies due to (informal) WPL during residency. Such self-awareness of competencies and knowledge can support self-efficacy, which has been mentioned as a further resource required for developing the intention to learn [11]. Self-efficacy refers to individual confidence in successfully handling challenging situations based on one’s own competencies [12, 13]. Among these factors, each resident’s motivation to gain a deeper understanding of a clinical topic, even outside of work, is important. We therefore assume that (informal) WPL plays a substantial role in the success of one’s residency [7, 14, 15].

Since the learning processes in residency training are less structured, but represent an innumerable variety of learning content and learning opportunities, which in turn can have a particular impact on patient care, we consider it urgently necessary to investigate the informal learning outcomes in residency training as well. Thus far, empirical research on informal WPL in residency training in general and on its outcomes in particular is scarce [16–20]. The present study addresses this gap by adapting the Questionnaire of Informal Workplace Learning Outcomes developed by Kyndt et al. [1] for use in residency training. Thus, we aim to answer the following research questions (RQ):

- RQ1: Does the German version of the instrument support a three-factorial model like the original instrument (cf. Kyndt et al. [1])?
- RQ2: Does the German version of the instrument provide reliable data (α ≥ .7)?
- RQ3: Is there a (predictive) relationship between medical expertise, communicative competence, and scholarship and informal WPL outcomes (*r* ≥ .3, *p* ≤ .05)?

## Method

### Residency in Germany

In Germany, residency training is independent from university-based undergraduate medical education. The medical associations (Ärztekammern) of the federal states are responsible for this, as they determine the learning content of specialist training. This includes mandatory sections in certain clinical areas (e.g. four years of anaesthesiology and one year of intensive care for an anaesthesiologist), as well as a correspondingly documented and performed minimum number of certain procedures (e.g. 50 central venous catheters). While the residents rotate through the different areas of their clinic to become familiar with the entire spectrum of their specialty, as well as to learn the necessary skills, the medical association decides at the end in a “collegial examination interview” whether to grant the specialist certification.

Currently, while competency-based curricula have been well integrated into undergraduate medical education, they are not generally applied to postgraduate specialist training. Furthermore, validated tools to assess competency do not yet exist or have not been accepted by German licensing institutions [21–23]. Although there is more competition for employment in the United States than in Germany, calls for changes in medical training have risen in recent years [24]. However, the structure of residency still differs substantially from that of American hospitals [25, 26]. In contrast, in countries like Great Britain and Ireland, for instance, competency-based assessments have been introduced to guarantee structured surgical training. These include specific teaching clinics, necessities for the ongoing education of the educators, and regular training days for trainees [27–29]. In Ireland, surgery residents need to complete lab-based operative skills assessments to be entrusted with professional activities [27, 29]. As mentioned above, German residency programmes may define the required content and length, but they do not emphasise the skills and competencies residents must possess at the end. Accordingly, the end of residency is defined by acquired time and knowledge, rather than skills and competencies.

### Sample

Data collection occurred in the context of a survey study conducted among Bavarian medical graduates (Bayerische Absolventenstudie Medizin, MediBAS) during October 2018 and January 2019. Invitations to participate were distributed among 1,610 physicians who graduated between October 1, 2016 and September 30, 2017 at one of five Bavarian medical schools (Friedrich-Alexander Universität Erlangen, Ludwig-Maximilians-Universität München, Technische Universität München, Universität Regensburg, and Julius-Maximilians-Universität Würzburg). The survey was distributed via mail (paper-based) or email (Questback, Globalpark Inc.; for more details, see [30]. A total of 528 participants (*n* = 339 female; age: *M* = 29.79, *SD* = 3.37 years) completed the survey and were included in the present study. Of these participants, 88.8% were still employed in their first job after graduation, 9.3% had already started another job, and only three physicians were not employed at the time of the survey. The average number of working hours per week was 52.37 (*SD* = 9.42, min = 8.50, max = 85.00). A total of 58.3% worked in institutions with more than 500 employees, 25.2% worked in hospitals with 50–499 employees, 11.4% worked in companies with 2–49 employees, and 5.1% did not provide any information. The distribution of medical disciplines can be found in Table 1, and the occupation type can be obtained from Table 2. At the time of the survey, the participants had been in graduate medical training for an average of 13.5 months (*SD* = 7.2). Participants who had not (yet) received graduate medical training were excluded from these analyses, as we could rule out WPL in residency training for these participants. The participants were informed about the content and purpose of the study and gave their informed consent to participate in the survey in advance. The data collection was anonymous.

**Table 1:** Distribution of medical disciplines among participants

Medical disciplines	*N*	%
Internal medicine	117	22.2
General medicine	66	12.5
Paediatrics	49	9.3
Anesthesiology	48	9.1
Orthopaedics and trauma surgery	33	6.3
Gynaecology and obstetrics	31	5.9
Neurology	26	4.9
Psychiatry and psychotherapy	24	4.5
Visceral surgery	17	3.2
Urology	14	2.7
General surgery	10	1.9
Otolaryngology	9	1.7
Ophthalmology	9	1.7
Radiology	8	1.5
Dermatology	8	1.5
Psychosomatic medicine and psychotherapy	6	1.1
Plastic and aesthetic surgery	4	0.8
Neurosurgery	4	0.8
Paediatric surgery	4	0.8
Child and adolescent psychiatry and psychotherapy	3	0.6
Vascular surgery	3	0.6
Public health	2	0.4
Radiotherapy	2	0.4
Pathology	2	0.4
Oral and maxillofacial surgery	2	0.4
Radiology	1	0.2
Physical and rehabilitative medicine	1	0.2
Nuclear medicine	1	0.2
Microbiology, virology, and infectious disease epidemiology	1	0.2
Human genetics	1	0.2
Occupational medicine	1	0.2
No decision	6	1.1
Others	8	1.5
No response	7	1.7

**Table 2:** Occupation type of participants

First occupation type	*N*	%
Clinical medicine with basic and standard care (care level I)	112	21.5
Clinical medicine, priority care (care level II)	134	25.4
Clinical medicine, university hospital/maximal care (care level III)	243	46.0
Resident physician	18	3.4
Social and health-related services	1	0.2
Private sector	1	0.2
University research	3	0.6
Others	10	1.9
No response	6	1.1

### Validation approach

Our validation approach is based on the construct validity concept, which postulates that the trustworthiness of a score can be determined by revealing the relationship between a (theoretical) construct and the measure under consideration through construct validity [31]. The first step in investigating construct validity was to examine the psychometric properties of the German translation of the original questionnaire. We started by examining the dimensionality (factor structure) of the scale using explorative and confirmatory factor analyses. This approach aimed to answer RQ1. We then examined the reliability of the instrument to answer RQ2.

Finally, we compared the results of the factor analysis with the three subscales of “medical expertise”, “communication”, and “scholarship” on the Freiburg Questionnaire to Assess Competencies in Medicine (FKM; [32]), for construct validity to answer RQ3. To investigate the construct validity of the WPL measure, we assume that a higher degree of competencies would be related to higher informal WPL outcomes.

### Instrument

The Questionnaire of Informal Workplace Learning Outcomes was developed and first validated for social care workers in Belgium [1]. A second Belgian study investigated informal WPL outcomes by adapting the instrument for policy inspectors [6]. Both studies applied the instrument in the Flemish language. The validation of the original instrument [1] consisted of three factors (GLO, OLLO, and JSLO) that were replicated in the study with policy inspectors [6]. The model fit in both studies was in an acceptable range ( [1]: CFI = 0.95; SRMR = 0.04, RMSEA = 0.05; [6]: CFI = 0.92; SRMR = 0.072, RMSEA = 0.078). Internal consistency was also satisfactory in both studies ( [1, 6]: α ≥ 0.73).

As the work environments of socio-educational care workers and healthcare providers are different, the original Questionnaire of Informal Workplace Learning Outcomes by Kyndt et al. [1] was adapted to the work environment of healthcare (see item wording in Table 3). Then, the questionnaire was translated into German. In translating the instrument, we followed the recommendations of Wild et al. [33]. The translation of the instrument was done by two educational researchers with English language skills at the C1–C2 level. A back-translation was not performed due to time constraints (fixed start of study from the administration committee of data collection). The translated version was discussed by the research group in order to ensure the substantive accuracy of the translation and its suitability to residency training. Based on the suggestions of Kyndt et al. [1], we adjusted the scale of the JSLO to our professional field of medicine. We eliminated item No. 10 on the original JSLO, “to support clients in their social participation”, as the item describes a (medical) activity that applies to only a small proportion of the medical profession (e.g. psychiatry or psychosomatics). As a result, we reduced the number of items from five to four. In addition, JSLO items No. 3 and No. 4 were adjusted to the medical context. The items were appraised on a 5-point Likert scale (1 = “disagree”, to 5 = “agree”).

**Table 3:** Overview of factor structure of measure by Kyndt et al., Janssens et al., and the present study

		Present study	Kyndt et al. 2013 [1]	Janssens et al. 2016 [6]
GLO_DW21	…to search for and make the most of opportunities, to take initiative by launching new ideas and taking action without waiting for others to take action	Generic learning outcomes: competence development	Generic learning outcomes	
GLO_DW25	…to form a thoroughly considered opinion and to undertake action and take responsibility for it at the right moment			Generic learning outcomes
GLO_DW22	…to estimate problems, hindrances, or opportunities in advance and to anticipate them			
GLO_DW17	…to develop my own talents and competences to achieve professional development			
GLO_DW2	…to acquire and process vocational information autonomously			
GLO_DW5	…to communicate orally and in writing with professionals about vocational topics			
GLO_DW23	…to proceed effectively in the treatment of patients in order to achieve maximum benefit with minimum effort			
GLO_DW18	…to optimise my own professional conduct based on reflection and feedback	Generic learning outcomes: reflection		
GLO_DW16	…to reflect autonomously, critically, and constructively on the functioning of my team, my colleagues, and the organisation			
GLO_DW19	…to reflect critically and constructively about my own professional conduct			
OLLO_DW15	…to pay attention to the broader context in which I work	Organisational learning outcome	Organisational learning outcome	Organisational learning outcome
OLLO_DW9	…to develop an understanding and involvement with regard to ethical, normative, and social questions			
OLLO_DW14	…to participate in policy development and policy implementation			
OLLO_DW11	…to fulfil managerial tasks autonomously			
JSLO_DW6	…to establish a therapeutic relationship with patients in order to provide them with necessary supports and services	Job-specific learning outcomes	Items adapted to occupational group	
JSLO_DW4	…to support patients cognitively, emotionally and therapeutically in their healing process in a respectful manner			
JSLO_DW10	…to develop therapeutic interventions in agreement with the patients in such a way that they are appropriate to the patients’ everyday lives			
JSLO_DW13	…to develop a treatment plan that addresses the patient’s specific medical problem and meets their needs			

The item wording are from the publication by Kyndt et al. [1], p. 12

Further data included age, gender, and the FKM score with the three subscales of “medical expertise”, “communication”, and “scholarship”*.* The scale measuring medical expertise referred to the knowledge and skills needed to conduct basic diagnostics and to develop treatment plans [32]. The scale for communication covers different communicative situations with patients and how to build trustworthy relationships with them [32]. Finally, the scale for scholarship covers the scientific competencies of reading, interpreting, and applying medical research findings in one’s daily work. The competencies described by the FKM are estimated on a 5-point Likert scale (1 = “not at all”, to 5 = “very much”; [32]).

### Analyses

Missing data were imputed using the Random Forest imputation method [34] in R by applying the R-packages “missForest” [35] and “randomForest” [36]. We started the instrument validation calculations with a CFA to answer RQ1. The aim of this CFA was to investigate the fit of the factor structure of the original scale developed by Kyndt et al. [1] for our data. Due to the poor data fit to the original model structure, we tested the common variance of the intercorrelation matrix using the Kaiser–Meyer–Olkin measure and Bartlett’s test of sphericity. The Kaiser–Meyer–Olkin measure of sampling adequacy provided a value of 0.91, and we found a significant result for Bartlett’s test (*p* < 0.001), showing that our data were suitable for exploratory factor analysis (EFA; [37]). We conducted an EFA with a robust maximum likelihood estimator to estimate the appropriate number of factors. Only variables with a loading ≥ 0.4 were considered in the respective factor [6, 37]. We then considered a four-factor solution for further analysis. The item OLLO No. 11, “…to fulfil managerial tasks autonomously”, did not fit any of the factors and was therefore excluded from further analyses.

We compared the two models with each other and found the most suitable structural equation model (SEM) using ANOVA. First, we compared the Akaike information criterion (AIC) and the Bayesian information criterion (BIC) of the two models. The smaller these values were, the better the model fit the data. Second, we interpreted the Chi^2^ differences and their significance. The Chi^2^ differences of the two models were significant (*p* < 0.001). Based on these comparisons, Model 1 performed better (Table 4). Then, we calculated the measurement models for each factor of Model 1 (cf. Table 5). The model fit values of each measurement model were found to be satisfactory [38]. For further analysis, we examined the explained variance for each factor and for the overall scale. The reliability of the factors was tested using McDonald’s omega and Cronbach’s alpha to answer RQ2. We assumed omega and alpha ≥ 0.7 to be adequate for a reliable test instrument [39].

**Table 4:** Comparison of fit indices of the two structural equation models

Model	X^2^ [df]	RMSEA	CFI	TFI	SRMR	AIC	BIC	X^2 Diff^ [df]^Diff^	*p-*Value
1	456.15 [113]	0.08	0.92	0.90	0.05	20,293	20,464		
2	712.59 [132]	0.09	0.86	0.84	0.06	22,015	22,181	256.44 [19]	< .001

Model 1: four-factorial model based on EFA; Model 2: factor structure of the original scale; X^2^ = chi-square (*p* < .001); df = degrees of freedom; RMSEA = root mean square error of approximation; CFI = comparative fit index; TFI = Tucker–Lewis index; SRMR = standardised root mean square residual; AIC = Akaike information criterion; BIC = Bayesian information criterion; X^2^ Diff = chi-square differences between Model 1 and Model 2; dfDiff = differences of degrees of freedom between Model 1 and Model 2

**Table 5:** Fit indices of measurement models of the four-factor solution

Items	*M*	*SD*	*X*^*2*^ [df]	RMSEA	CFI	TFI	SRMR	Ω [ 95% CI]	Alpha [ 95% CI]
GLO-CD	3.56	.67	91.44 [14]	0.10	0.94	0.91	0.043	.85 [.83,.87]	.85 [.83,.87]
GLO_DW21	3.11	.98							
GLO_DW25	3.54	.89							
GLO_DW22	3.57	.82							
GLO_DW17	3.61	.98							
GLO_DW2	3.69	.90							
GLO_DW5	3.80	.91							
GLO_DW23	3.63	.95							
GLO-R	3.92	.74	0.00 [0]	0.00	1.00	1.00	0.000	.84 [.80, .85]	.83 [.80, .85]
GLO_DW19	3.99	.85							
GLO_DW18	3.90	.92							
GLO_DW16	3.87	.80							
JSLO	3.78	.76	35.71 [2]	0.18	0.96	0.88	0.040	.84 [.82, .86]	.84 [.82, .86]
JSLO_DW6	3.98	.89							
JSLO_DW4	3.98	.93							
JSLO_DW10	3.64	.93							
JSLO_DW13	3.48	.96							
OLLO	3.57	.84	0.00 [0]	0.00	1.00	1.00	0.000	.69 [.64, .74]	.69 [.64, .74]
OLLO_DW15	3.54	.96							
OLLO_DW19	3.65	.94							
OLLO_DW14	2.89	1.09							
Freiburg Questionnaire to Assess Competencies in Medicine [21]									
Communication	2.92	.80						.93 [.93, .94]	.93 [.93, .94]
Medical Expertise	3.36	.59						.88 [.87, .90]	.89 [.87, .90]
Scholarship	2.75	.80						.93 [.92, .94]	.93 [.92, .94]

*M *Mean, *SD *Standard deviation, X^*2 *^chi-square, *df *degrees of freedom, *RMSEA *Root mean square error of approximation, *CFI *Comparative fit index, *TFI*  Tucker–Lewis index, *SRMR *Standardised root mean square residual, Ω McDonald’s omega; overall factor mean (SD) see Table 3; original item wording in English published by 1 [1]

To answer RQ3, we then investigated the relationship between the WPL factors and the three FKM scales of “medical expertise”; “communication”, and “scholarship” using Spearman’s rho correlation coefficient due to a missing normal distribution of the data. According to the work of Field [40], we assumed a correlation coefficient of *r* ± 0.1 as a small effect, *r* ± 0.3 as a medium effect, and *r* ± 0.5 as a large effect. The significance level was set at *p* ≤ 0.05. The analyses were conducted with the R package lavaan (version 3.6.1; [41, 42]) and IBM SPSS statistical software (version 28; [43])*.*

To further investigate the construct validity of the instrument, we calculated an SEM by applying the three FKM scales as predictors of the four WPL factors. In the first step, a measurement model was calculated that covered the four latent variables of informal WPL outcomes. In the second step, a regression model was conducted to add the three FKM scales as predictors to the measurement model [39]. The SEM was calculated by using the software M*plus* 8.7 [44] by applying maximum likelihood. The cut-offs of the fit indices for SEM applied by Kline [39] were used here (CFI ≥ 0.90, TFI ≥ 0.95, SRMR ≤ 0.05; RSMEA ≤ 0.08).

## Results

### Factor analysis

To answer the first research question regarding whether the German version of the instrument supported the same factor structure as the original instrument, we conducted a CFA. All items were modelled to load onto the respective factors in accordance with the original scale [1]. In contrast to the original scale, the model fit indices were poor (cf. Table 4, Model 2). We did not find a factor structure similar to that of the original scale as postulated in RQ1. After the CFA, we investigated the factor structure using EFA. The EFA revealed a four-factor solution as the best fit for our data (Model 2).

### Reliability

The second research question focused on the reliability of the factors within the measure, which was calculated using McDonald’s omega. The values of all four factors were in an acceptable range, thereby providing evidence of good internal consistency (α ≥ 0.69, Ω ≥ 0.69; cf. Table 5).

### Correlation and regression analyses

The test of normal distribution of the factor means and the means of communication competence, scholarship, and medical expertise were significant. Therefore, we could not assume normally distributed data. To investigate the construct validity of the scale, we calculated an SEM with the four factors of the informal WPL outcome questionnaire and the three scales of the FKM, including “communication”, “scholarship” and “medical expertise” (cf. Table 6). Spearman’s rho correlations between the factor means and the three competency means of “communication”, “scholarship”, and “medical expertise” were of medium size (between *r* = 0.199 and *r* = 0.395). Therefore, we could assume that the four factors of the WPL questionnaire were related to the three competencies of communication, medical expertise, and scholarship, indicating comparable but different underlying constructs. In addition, we investigated whether the three perceived competencies predicted informal WPL outcomes by applying an SEM. The SEM indicated that medical expertise in particular had an impact on all four factors of the informal WPL outcomes measure. In contrast, scholarship seemed to predict GLO-CD and GLO-R, and communication had no predictive effect on any of the four factors (Table 7).

**Table 6:** Correlation matrix between the four factor means and communication, scholarship, and medical expertise of the FKM

	GLO-R	JSLO	OLLO	Communication	Scholarship	Medical expertise
GLO-CD	.563	.580	.509	.328	.349	.395
GLO-R		.461	.478	.219	.222	.219
JSLO			.552	.199	.188	.261
OLLO				.247	.247	.321
Communication					.493	.588
Scholarship						.546

All correlations are significant, *p* < .001

**Table 7:** Regression of the four WPL factors on communication, scholarship, and medical expertise

Predictors	GLO-CD			GLO-R			JSLO			OLLO		
	*B*	*ß*	*p*	*B*	*ß*	*p*	*B*	*ß*	*p*	*B*	*ß*	*p*
Communication	0.080	0.102	.067	0.054	0.060	.312	0.077	0.088	.133	0.027	0.036	.555
Scholarship	**0.139**	**0.175**	**.001**	**0.122**	**0.135**	**.017**	0.065	0.075	.185	0.084	0.111	.063
Medical expertise	**0.260**	**0.242**	** < .001**	**0.151**	**0.123**	**.046**	**0.207**	**0.174**	**.005**	**0.337**	**0.328**	** < .001**

*Ꭓ*^*2*^ (152) = 495.27, *p* < .001; RMSEA = 0.065; CFI = 0.918; SRMR = 0.044; R^2^: F1 = 0.193, F2 = 0.072, F3 = 0.083, F4 = 0.180; *B* = unstandardised regression coefficients; *ß* = standardised (stdyx) regression coefficient

## Discussion

Our study examined the psychometric properties of the adapted German version of the Questionnaire of Informal Workplace Learning Outcomes [1] for medical residency. Furthermore, we examined how the questionnaire was related to the residents’ perceived competencies of medical expertise, scholarship, and communication [32]. In the following sections, we discuss the answers to our research questions and the limitations and practical implications of our study.

The first research question investigated whether the German version of the questionnaire, when adapted for medical residency, supported the three-factorial model of the original instrument by applying a CFA (cf. [1]). In this case, the CFA provided insufficient model-fit values. One explanation could be that the reference frame with which the doctors completed the questionnaire was different from that used by social care workers in the original questionnaire [1] or by policy inspectors in an additional adaptation of the instrument [6]. Thus, the items may have been scored differently than in the original study. This, in turn, explains the different scale formation, since the outcomes of a consecutive EFA suggested a four-factor model with acceptable fit values. In comparison to the original three-factorial model (see Table 3), our four-factorial model also included the JSLO and OLLO factors. The GLO-CD and GLO-R depicted different aspects of generic learning outcomes. The GLO-CD included items about competence development, and the GLO-R emphasised items that describe reflective processes of (generic) learning. The distinction between job-specific and GLO was an adaptation of the original classification of items by Kyndt et al. [1]. Below, we briefly discuss each factor.

The GLO-CD, referring to generic learning outcomes—competence development*,* included items that described generic learning outcomes with a focus on competence development. We considered this to be an important dimension, as it reflects one’s cognitive independence and freedom, for which some critical thinking and some cognitive spare capacity might be prerequisites. Furthermore, this perspective on competence development could refer to the aspect of becoming a physician during residency. Following the idea of professional identity formation, gathering experiences should facilitate identity formation and allow for a transition from imitating the physician’s role to being a physician [45]. This factor extends to some extent to the professional identity formation of becoming an independent and autonomous thinking and acting physician who is able to break the boundaries of their profession in an appropriate manner [46].

The GLO-R, or generic learning outcomes—reflection, consisted of items that described reflection processes in the occupational environment. Reflection is a crucial part of learning in general and of WPL in particular. Through reflection, trainees can learn from their experiences and transfer their learning to comparable future situations [47]. The aspect of reflection is also described in the Canadian Royal College of Physicians and Surgeons’ physician competency framework (CanMEDS) with respect to the role of the “professional”, where the physician’s commitment to self includes self-monitoring to constantly improve their behaviour; this is relevant to both how they treat patients and how they manage their own resources [48]. In the field of expertise development, the concept of deliberate practices also refers to reflection as a crucial skill for improving one’s competencies and performance [49]. These different excerpts are only a brief indication of the manifold relevance of reflection in undergraduate medical education and residency training, and support the assumption that reflection also represents a crucial aspect of informal WPL in residency training [6].

The JSLO, or job-specific learning outcomes, focused on interpersonal relationships with the patient and effective, informed patient treatment. A good doctor-patient relationship facilitates a patient’s compliance and a physician’s job satisfaction and reduces the physician’s distress and burn-out risks [50]. Aspects of this factor are also covered in CanMEDS’ roles of the “communicator” and “collaborator”, where the communicator facilitates the doctor-patient relationship and shared decision making, and the collaborator fosters a successful relationship “with other health care professionals to provide safe, high-quality, patient-centred care” [48]. Given that patient encounters are central to the medical profession [51], and also represents a plausible facet of informal WPL outcomes.

OLLO accounts for organisational-level learning outcomes. This factor is comparable to the original factor introduced by Kyndt et al. [1]. OLLO covers the general aspects of a professional occupation that can be applied regardless of the specific work domain. Specifically, these include competencies for taking organisational and social responsibility, such as developing and implementing new ideas, considering one’s activities in the larger context, and dealing with ethical and social issues [1, 52]. These aspects of informal WPL outcomes seem to be transferable across different disciplines and play a crucial role in the medical field, as well as in social care or policy work environments [1, 6].

Finally, the CFA provided acceptable model fit values and showed comparable relationships between the factors, like the original instruments. The results support construct validity and indicate that the four factors support a similar construct [1, 39].

Our second research question aimed to examine the reliability of the instrument. Good internal consistency supports the assumption that the items measure the same underlying construct, which is also a prerequisite for a latent-variable analysis, like applying SEM [39], and for interpreting the validity of the measure [53]. All four factors provided good internal consistency. Compared to the original scale and the adaptation for police inspectors, similar internal consistency was measured. Therefore, we assume a reliable measure, which allowed for the further assumption that the content of the items of the subscales was homogeneous [39].

According to the results of our first and second research questions, we investigated our third research question on whether there was a relationship between the responses for informal WPL outcomes and the three competences of medical expertise, scholarship, and communication. Our study shows the relationships between these three competences and the four factors of the informal WPL outcomes measure. These correlations provide support for the assumption of construct validity [31, 39]. Applying the three competences as predictors of the informal WPL outcomes shows that “medical expertise” predicts the informal WPL outcomes of all four factors of the measure. This seems plausible due to the necessity of building baseline knowledge and skills to conduct anamnesis and to develop diagnoses and appropriate treatment plans [32, 48]. In addition, the predictive characteristics of scholarship for the two generic learning factors are plausible due to the ability to identify learning needs and to generate new knowledge [32, 48, 54]. The concept of scholarship contains the abilities of reading, interpreting, and applying scientific medical findings to the daily work at ward and to the particular patient’s needs and concerns [32], as well as to lifelong learning [48]. Communication had no predictive effect on any factor. The missing predictive relationship between communication and informal WPL outcomes could be due to the different aspects of the two measures. The communication factor of the FKM refers only to communication with patients and within teams, whereas the WPL factors refer more to the broader aspects of professional behaviour in the clinical setting and to skills such as scientific communication, conflict management, and giving and asking for feedback. Previous research has provided evidence that asking for and giving feedback are related to informal WPL outcomes [6, 55]. While the team communication aspect of the FKM communication scale could indicate a positive correlative relationship between communication and informal WPL outcomes, this does not explain why communicative competence predicts informal WPL outcomes.

The results pertaining to the relationship between informal WPL outcomes and the three medical competencies are in line with previous research on the antecedents of workplace learning, where the level of competence or basic-level skills relate to WPL outcomes [11, 55]. Our research findings thus provide initial exploratory results confirming that the informal WPL outcomes measure can provide appropriate information about different learning outcomes and their antecedents during residency.

While interpreting the data, we had to consider some limitations of our study. First, we used a convenience sample. We asked only graduates of one year in one federal state to participate via mail and email. Therefore, motivation and interest in medical education might have had an impact on their participation, and we cannot preclude the possibility that participants did not differ significantly from non-participants on these points. Second, competence assessments are based on self-assessments, which can be influenced by different biases. For instance, graduates have been shown to overestimate their abilities more often, especially those with lower competencies [56, 56]. In this case, the competencies of medical expertise, scholarship, and communication refer to the perceived competencies at the end of undergraduate studies, so a distorted perception through time might be plausible [57]. Furthermore, the recalling of experiences often refers to selective situations instead of all-day situations; therefore, the participants might not be able to accurately remember their demonstrated competencies and learning experiences in the workplace. These circumstances might influence the relationship between perceived competencies and WPL outcomes. Future studies could therefore relate the assessments of work samples to informal WPL learning outcomes to substantiate the validation process of the measure.

The practical implementation of our work here still has the characteristics of a research study. Our study is the first attempt to apply and validate this particular measure in (graduate) medical education. Our study results thus contribute to the research on learning conditions in residency training. However, to date, no recommendations or conclusions can be made about “good” WPL at the personal or organisational level. Further research on the predictors of WPL in residency training is thus needed to draw conclusions about the quality of WPL through the application of this measure.

## Conclusion

This study provided evidence to validate the use of an existing instrument to assess informal WPL outcomes during residency. Although we were unsuccessful in replicating the three-factor model of the original questionnaire, our four-factor model nonetheless revealed meaningful factors of informal WPL related to residency training. The four factors were predicted by the competence facets of medical expertise and scholarship.

We are convinced that the tool is critical for identifying points for action and structural improvement with respect to the non-technical aspects of learning during residency. A longitudinal perspective should also be considered to examine learning outcomes during residency years.

## Data Availability

Since data sharing was not included in the proposal approved by the Ethics Committee, these data cannot be shared. However, the data are available upon reasonable request from the corresponding author.

## Abbreviations

AIC: Akaike information criterion
ANOVA: Analysis of variance
BIC: Bayesian information criterion
CFA: Confirmatory factor analysis
EFA: Exploratory factor analysis
FKM: Freiburg Questionnaire to Assess Competencies in Medicine
GLO: Generic learning outcomes
GLO-CD: Generic learning outcomes - competence development
GLO-R: Generic learning outcomes - reflection
JSLO: Job-specific learning outcomes
MediBAS: The survey of Bavarian medical graduates
OLLO: Organisational-level learning outcomes
RQ: Research question
SEM: Structural equation model
WPL: Workplace learning
